# Resection of Mycotic Iliac Artery Aneurysm with Extra-Anatomic Bypass: An Alternative to Aneurysmorrhaphy in Difficult Situations

**DOI:** 10.21470/1678-9741-2023-0350

**Published:** 2024-07-15

**Authors:** Vikas Deep Goyal, Gaurav Misra, Akhilesh Pahade, Neeraj Prajapati

**Affiliations:** 1 Department of Surgery, Shri Ram Murti Smarak Institute of Medical Sciences, Bareilly, Uttar Pradesh, India; 2 Department of Anesthesia, Shri Ram Murti Smarak Institute of Medical Sciences, Bareilly, Uttar Pradesh, India; 3 Department of Radiodiagnosis, Shri Ram Murti Smarak Institute of Medical Sciences, Bareilly, Uttar Pradesh, India

**Keywords:** Infected Aneurysm, Morbidity, Arteries, Abdominal Aortic Aneurysm

## Abstract

Mycotic aneurysms of the iliac and other large arteries are rare and are associated with increased morbidity and mortality. Treatment of mycotic aneurysms usually requires modification of the surgical technique done for cases of degenerative or atherosclerotic aneurysms. Degenerative and atherosclerotic fusiform aneurysms are usually managed with aneurysmorrhaphy using a prosthetic graft, which however is not ideal for mycotic aneurysms. Avoidance of prosthetic material at the site of mycotic aneurysm is a better option with higher chances of resolution of infection and favorable patient outcome.

## INTRODUCTION

The etiology of mycotic aneurysms includes iatrogenic vascular injury, intravenous drug abuse, endocarditis, osteomyelitis, bacteremia due to any cause, and idiopathic causes^[1]^. Arteries usually affected by mycotic aneurysms are the femoral artery, thoracic aorta, abdominal aorta, and popliteal artery; other arteries including iliac arteries are rarely involved^[1]^. Mycotic aneurysms of the aorto-iliac segment are rare and have a high mortality rate^[2]^. *Staphylococcus, salmonella*, and *Streptococcus* bacteria have been more commonly isolated from patients with mycotic aneurysms^[2,3,4]^. The treatment of fusiform non-mycotic atherosclerotic/degenerative aneurysm is usually done with either aneurysmorrhaphy or endovascular interventions. There are situations where aneurysmorrhaphy is not a suitable option or endovascular intervention is technically difficult. The presence of mycotic aneurysms or intraoperative presence of unhealthy and infected tissue requires resection of the aneurysm and reconstruction with an autologous tissue either in an extra-anatomic route or by *in situ* placement. *In situ* placement of the graft is usually required for reconstruction of the thoracic or abdominal aorta; for reconstruction of the iliac arteries, extra-anatomic bypass is a suitable alternative^[4,5]^.

A case of a large mycotic left common iliac artery (CIA) aneurysm with significant comorbidities is discussed. Written informed consent was taken from the patient for publication of the clinical data.

## CASE PRESENTATION

**Fig. 1:**
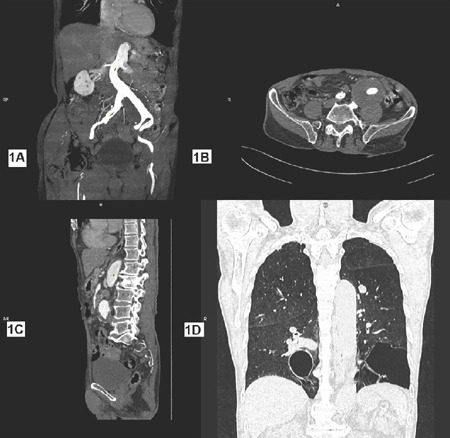
1A) Computed tomography (CT) angiogram coronal section shows large aneurysm involving left common iliac artery; 1B) CT angiogram axial section shows partially thrombosed aneurysm; 1C) CT angiogram sagittal section; 1D) CT chest showing large bullae in the lower lobe of both lungs.

A 68-year-old man presented with pain and mass in the abdomen for three months. His symptoms were progressively increasing. The patient was a chronic smoker for the last 35 years and had developed chronic obstructive pulmonary disease (COPD). On examination, the mass was in the periumbilical region and extended into the left iliac fossa. The mass was pulsatile and there was bruit audible over the mass. Pulsations in the left lower limb were feeble as compared to the right lower limb. On auscultation of the respiratory system, rhonchi were present and air entry was decreased in the lower lobes more on the left side. Computed tomography (CT) angiogram of the abdominal aorta revealed a large left CIA aneurysm arising just distal to the aortic bifurcation with atherosclerotic changes in the abdominal aorta and both iliac vessels (Figure 1A, 1B, and 1C). High-resolution CT of the chest revealed large bullae in the lower lobes of both lungs (Figure 1D). The patient also had moderate left ventricular (LV) dysfunction with ejection fraction (EF) of 35% and regional wall motion abnormalities (RWMA). Intervention radiology consultation was taken for endovascular intervention, but the patient was referred back to us citing unfavorable anatomy. Acute angulation at the bifurcation of the left CIA into the left external iliac artery and left internal iliac artery along with diffuse atherosclerosis were the reasons for the unfavorable anatomy for endovascular intervention. Surgical intervention was thereafter planned.

### Surgical Treatment

**Fig. 2:**
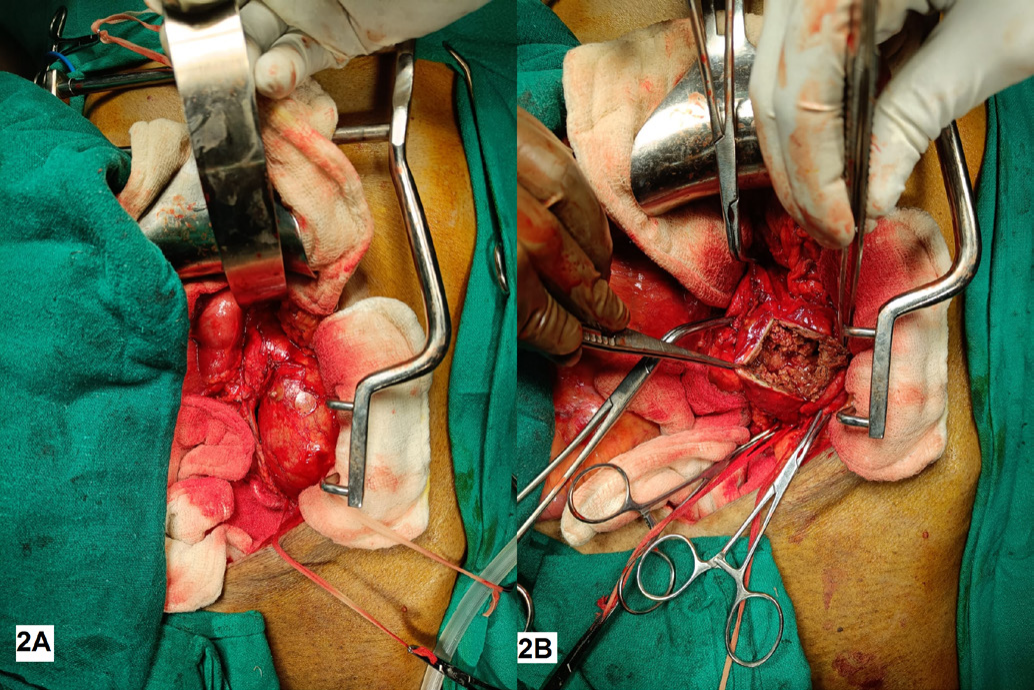
2A) Operative image shows the aneurysm in the left common iliac artery just distal to the aortic bifurcation; 2B) operative image of the opened-up image of the thin-walled aneurysm with clots and infected tissue.

**Fig. 3:**
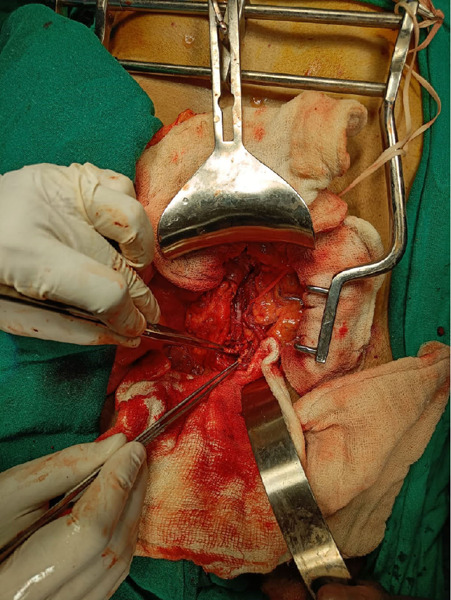
Operative image showing ligated stumps of the left external and internal iliac arteries and repaired aorta in continuation with right common iliac artery after resection of the mycotic aneurysm.

Coronary angiography was advised to the patient, but the patient was unwilling for any coronary intervention prior to the treatment of the aneurysm. Cardiologist opinion was also taken in case, urgent coronary angioplasty is required anytime during the perioperative period. Surgical intervention in the form of aneurysmorrhaphy using a prosthetic Dacron® graft was planned. As the patient had severe COPD and bilateral lower lobe bullae in the lungs, general anesthesia was considered risky, fearing rupture of the bullae with positive pressure ventilation. Therefore, the patient was planned for surgical intervention under combined spinal and epidural analgesia. The surgical approach was through midline laparotomy starting just above the umbilicus to above the pubic symphysis. The infrarenal aorta was exposed along with control of bilateral iliac arteries. The left ureter was mobilized from the aneurysm wall and looped away from the surgical field. Distal control in the left internal iliac and left external iliac was also taken. After heparinisation (1 mg/kg), clamps were applied on the infra-renal aorta, left external iliac, left internal iliac artery, and right CIA (Figure 2A). The aneurysm was then opened, and to our surprise, dirty fluid came out, which was sent for culture sensitivity. Aneurysm contained large amounts of macerated clots, and the aneurysm wall was thinned out (Figure 2B). Intraoperatively, we decided to do aneurysm resection along with extra-anatomic bypass instead of aneurysmorrhaphy. The aneurysm wall was dissected from the surrounding tissue and most of it was excised. The area was copiously washed with normal saline. The proximal ends of the left external iliac artery and left internal iliac artery were trimmed till healthy tissue was reached and closed with running polypropylene sutures (4'0'). The proximal end of the left CIA was transected, and the aorta was repaired using continuous polypropylene sutures without leaving a stump protruding from the aorta. The infrarenal aorta thereby continued as the right CIA (Figure 3). After hemostasis, bilateral femoral arteries were exposed in the groin through vertical incisions. Saphenous vein graft was harvested from the right thigh; a subcutaneous tunnel in the suprapubic area was created for the vein graft with blunt dissection well below the midline incision. Crossover femoro-femoral bypass was done using reversed saphenous vein graft. Leg and thigh incisions were closed after hemostasis. A pelvic drain was placed, and the abdominal incision was then closed after ensuring hemostasis at the suture line in the abdominal aorta and the stumps of left external iliac and internal iliac arteries. Pulsations in both lower limbs were present post-procedure. Total blood loss during the procedure was < 300 ml. Abdominal drain was removed on the second postoperative day, and the patient was discharged on the seventh postoperative day. The patient came for a follow-up 15 days after the procedure and was doing well with palpable pulsations in both lower limbs. The cultures were sterile, but the biopsy was suggestive of mycotic aneurysm. The patient was then referred to cardiologist for coronary angiography.

## DISCUSSION

Mycotic aneurysms of major arteries are associated with high mortality and morbidity^[4,5]^. The options available for such cases include: 1) resection of the aneurysm with all the infected tissue and extra-anatomic bypass with autologous graft or prosthetic graft (femoro-femoral bypass/axillo-femoral bypass); 2) resection of the aneurysm with all the infected tissue and *in situ* reconstruction with autologous grafts like femoro-popliteal vein or cryopreserved allograft; 3) endovascular interventions.

Thoracic and suprarenal mycotic aneurysms are usually managed with *in situ* grafting, whereas for infrarenal and iliac aneurysms extra-anatomic bypass is usually done apart from resection of the infected aneurysmal segment^[4,5]^. *In situ* grafting for large vessels can be done with a segment of femoro-popliteal vein or with a cryopreserved allograft to avoid prosthetic graft^[6,7]^. Recent reports in the literature have reported favorable results with the endovascular intervention^[8]^.

Smoking is a risk factor for aneurysmal disease as well as coronary artery disease and COPD. All these comorbidities were present in this case. Preoperative assessment for all these diseases helps in intraoperative planning and may require modification of the anesthetic technique. Diagnosis of large lung bullae preoperatively in this case helped us to modify the anesthetic technique to combined spinal and epidural analgesia instead of general anesthesia with positive pressure ventilation, thereby avoiding the risk of pneumothorax/tension pneumothorax in the perioperative period. The presence of RWMA along with moderate LV dysfunction increases the risk of acute coronary events and the involvement of a cardiologist in the perioperative period is also helpful.

## CONCLUSION

Isolated iliac artery aneurysm surgery can be safely performed under combined spinal and epidural analgesia by taking adequate precautions in high-risk patients with significant comorbidities. Resection of the aneurysm with extra-anatomic bypass is a suitable alternative to aneurysmorrhaphy for patients having mycotic aneurysm. Combined spinal and epidural analgesia may be more suitable than general anesthesia and positive pressure ventilation in patients with large bullae in lungs. Improvements in surgical and anesthetic techniques have improved the results of surgery in such cases with significant comorbidities.

## Abbreviations, Acronyms & Symbols

CIA: Common iliac artery
COPD: Chronic obstructive pulmonary disease
CT: Computed tomography
EF: Ejection fraction
LV: Left ventricular
RWMA: Regional wall motion abnormalities
